# From Spark to Flame: ROS‐ and Light‐Cascade Activatable NIR‐II AIE Probe for Precise Tumor Imaging and Self‐Amplifying Phototherapy

**DOI:** 10.1002/advs.202514789

**Published:** 2025-11-02

**Authors:** Xiaohui Chen, Yuanyuan You, Songling Lin, Chengwei Tang, Jun Zhu, Qiongwen Liang, Dan Rao, Jiali Deng, Yuxun Ding, Dingyuan Yan, Wenman Li, Dong Wang, Ben Zhong Tang

**Affiliations:** ^1^ School of Medical Technology Guangdong Medical University Dongguan 523808 China; ^2^ School of Pharmacy Guangdong Medical University Dongguan 523808 China; ^3^ Center for AIE Research Shenzhen Key Laboratory of Polymer Science and Technology College of Materials Science and Engineering Shenzhen University Shenzhen 518060 China; ^4^ Guangdong Basic Research Center of Excellence for Aggregate Science School of Science and Engineering The Chinese University of Hong Kong Shenzhen Guangdong 518172 China

**Keywords:** aggregation‐induced emission, cancer phototheranostics, NIR‐II emission, photoactivation

## Abstract

The development of photoactivatable theranostic probes represents a major focus in precision tumor therapy. However, those previously reported probes often suffer from limited photoresponsivity, short excitation/emission wavelengths, and inactivity in the absence of light, restricting their ability to precisely diagnose deep‐seated tumors or enable effective phototherapy without auxiliary interventions. To address these challenges, this study designs a second near‐infrared (NIR‐II) aggregation‐induced emission (AIE) theranostic probe based on a dihydroindole skeleton, featuring dual reactive oxygen species (ROS)‐ and NIR light‐cascade activation. Upon ROS activation in the tumor microenvironment, TT‐DHIn undergoes transformation into TT‐In, exhibiting NIR‐II fluorescence emission and photodynamic/photothermal therapy (PDT/PTT) capabilities, thereby serving as a photoactivatable “guiding radar” with an exceptional signal‐to‐background ratio. Following pre‐activation, TT‐In efficiently generates ROS under 660 nm laser irradiation, enabling self‐supplementation of intratumor ROS. Furthermore, the intratumor TT‐DHIn undergoes cyclic conversion into TT‐In, significantly enhancing phototherapeutic efficacy and demonstrating potent in vitro cytotoxicity and in vivo tumor eradication. This dual‐activatable cascade strategy synergistically integrates tumor biomarker (ROS) responsiveness with photoactivation, offering a promising platform for NIR‐II imaging‐guided precision phototheranostics with strong potential for clinical translation.

## Graphical abstract

ROS‐ and NIR light‐cascade activatable NIR‐II AIE theranostic probe (TT‐DHIn) is developed by integration of dihydroindole skeleton with oxidative dehydrogenation reaction mechanism and AIE skeleton with a twisted configuration. Upon ROS in the tumor microenvironment and NIR light activation, TT‐DHIn can achieve dual‐model imaging‐guided precise tumor therapy with the philosophy of “pre‐localizing tumor, then self‐amplifying phototherapy”.

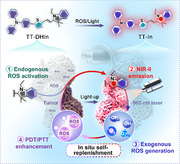

## 1 Introduction

The exploration of activatable theranostic agents exhibits inexhaustible and vigorous vitality in the field of precision medicine,^[^ ^1^, ^2^, ^3^, ^4^ ^]^ of particular interest is the counterparts mediated by light irradiation,^[^ ^5^ ^]^ mainly benefiting from the inherent superiorities of those photoactivatable theranostic protocols, such as non‐invasiveness, remarkable spatiotemporal controllability, high signal‐to‐background ratio (SBR), and minimal side effects.^[^ ^6^, ^7^, ^8^ ^]^A series of photoactivatable fluorescent probes based on individual photochemical reaction mechanisms have been recently developed for bioimaging and therapy.^[^ ^9^, ^10^, ^11^, ^12^, ^13^ ^]^ The photoactivatable probes always remain non‐emissive and therapeutically inert at the tumor location prior to light irradiation,^[^ ^14^, ^15^, ^16^ ^]^ which precludes the pre‐diagnosis of tumor lesions via fluorescence imaging techniques, thereby impeding effective tumor phototherapy. Thus, improving the tumor phototherapeutic efficacy can only be performed by pre‐locating tumor with the assistance of external imaging technologies, such as computed X‐ray tomography,^[^ ^17^ ^]^ magnetic resonance imaging,^[^ ^18^ ^]^ positron emission tomography,^[^ ^19^ ^]^ etc. However, they suffer from inherent and collective defects, including poor spatiotemporal resolution, low sensitivity, radiation hazards, and long signal collection time, which seriously restrict the application of photoactivatable probes for in vivo tumor therapy.^[^ ^20^, ^21^ ^]^

On the other hand, the excitation light sources of most photoactivatable probes are usually high‐energy ultraviolet or visible light (400–700 nm),^[^ ^22^, ^23^, ^24^ ^]^ and their emission wavelengths are commonly located in the visible and first near‐infrared (NIR‐I, 700–900 nm) regions,^[^ ^25^, ^26^ ^]^ resulting in shallow tissue penetration depth, strong biological tissue absorption and scattering, fluorescence self‐quenching in the aggregate state, etc.^[^ ^27^, ^28^ ^]^ These challenges severely restrict their extensive clinical transformation.^[^ ^29^, ^30^ ^]^ Fortunately, second near‐infrared (NIR‐II, 1000–1700 nm) fluorescence imaging has broad application prospects in tumor diagnosis by virtue of its advantages, including high tissue penetration depth, low tissue absorption, and excellent SBR.^[^ ^31^, ^32^, ^33^, ^34^ ^]^ Moreover, benefiting from the excellent photophysical properties, aggregation‐induced emission luminogens (AIEgens) can perfectly address the issue of fluorescence self‐quenching and low phototherapy in an aggregate state.^[^ ^35^, ^36^, ^37^, ^38^ ^]^ Therefore, the development of AIE‐active NIR‐II theranostic probes that integrate tumor‐localization visualizing function with photoactivation capability is expected to break through the bottleneck that photoactivatable probes themselves cannot achieve tumor diagnosis and treatment.

Tumor microenvironment (TME) has higher reactive oxygen species (ROS) levels than that of normal tissue.^[^ ^39^, ^40^ ^]^ ROS‐activatable fluorescent probes can specifically generate the light‐up fluorescence signals at the tumor location, enabling precise tumor diagnosis.^[^ ^41^, ^42^, ^43^ ^]^ Until now, a series of ROS‐activatable NIR‐II fluorescent probes for tumor localization have been developed,^[^ ^44^ ^]^ such as Ag/Ag_2_S Janus nanoparticle,^[^ ^45^ ^]^ CuS‐ and Ag_2_S‐based intelligent nanofactory,^[^ ^46^ ^]^ thiophene‐based amphiphilic NIR‐II small molecule,^[^ ^47^ ^]^ phenylboronic acid ester fragment‐based BODIPY derivative,^[^ ^48^ ^]^ urea bond‐based leucomethylene derivative,^[^ ^49^ ^]^ luminol‐based NIR‐II molecule,^[^ ^50^ ^]^ and so forth. It has been demonstrated that the intratumor ROS exhibit substantial heterogeneity and significantly spatiotemporal variations, which can only lead to partial activation of the probes.^[^ ^51^, ^52^, ^53^ ^]^ Therefore, utilizing intratumor ROS‐activatable probes to generate exogenous ROS upon light irradiation, which subsequently trigger the activation of unactivated probes, would amplify phototherapy outcomes and consequently achieve effective tumor theranostics.

**Scheme 1:**
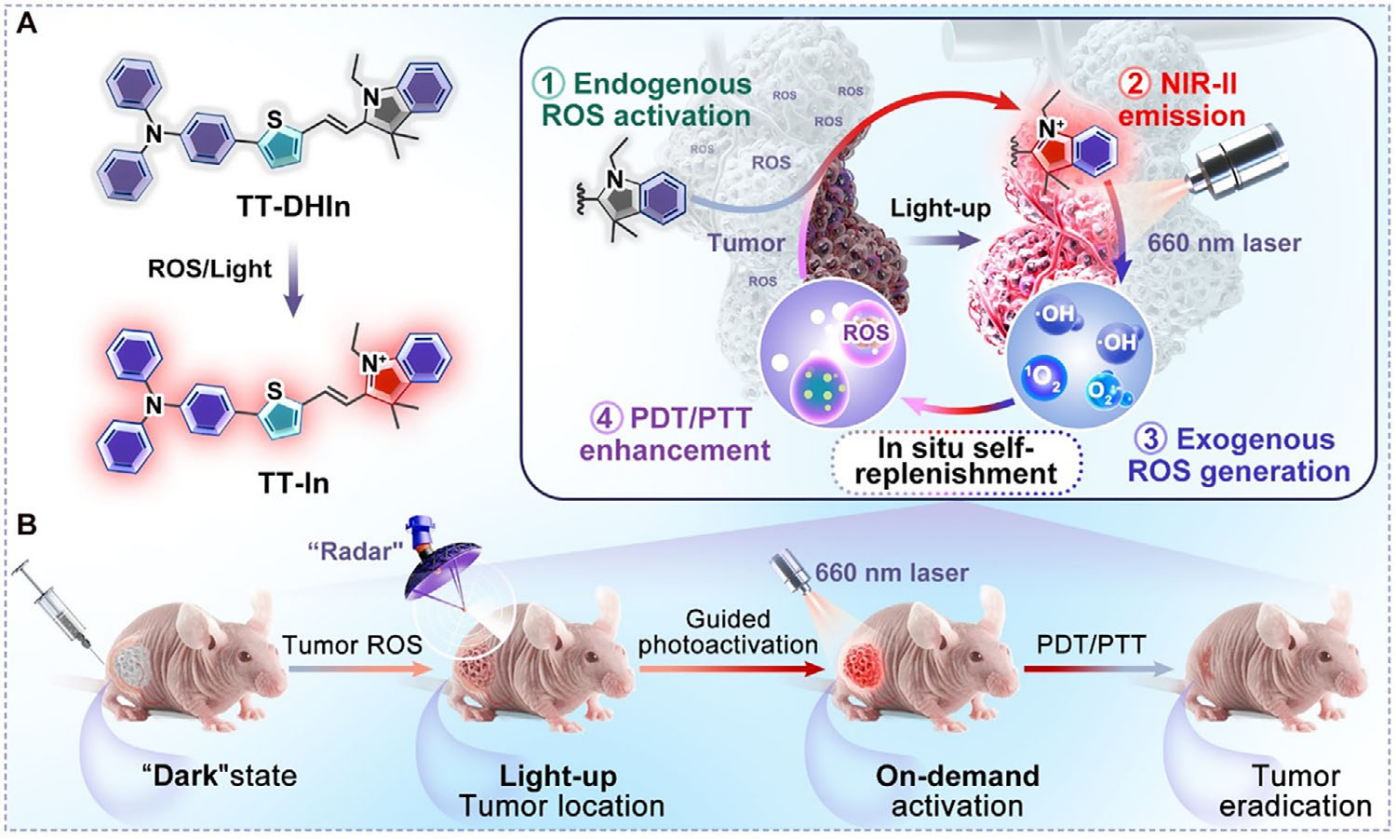
Schematic illustration of ROS‐ and light‐cascade activatable NIR‐II AIE probe for precise cancer phototheranostics.

Herein, we developed a ROS‐ and light‐cascade activatable NIR‐II AIE theranostic probe, namely TT‐DHIn, bearing the dihydroindole (DHIn) skeleton with oxidative dehydrogenation reaction mechanism and the AIE skeleton with a twisted configuration for the imaging‐guided precise tumor therapy with the philosophy of “pre‐localizing tumor, then self‐amplifying phototherapy” (**Scheme** **1**). TT‐DHIn could rapidly convert into the corresponding indolium salt counterpart TT‐In via ROS‐induced oxidative dehydrogenation reaction, and with the assistance of laser irradiation, the generated NIR‐II fluorescence signals of TT‐In can be used to pre‐localize tumor. Synchronously, targeted laser irradiation is capable of effectively triggering ROS production of TT‐In in the tumor, consequently achieving self‐replenishment of ROS in the TME and effective activation of the TT‐DHIn probe, significantly amplifying the phototherapeutic activity.

## 2 Results and Discussion

### 2.1 Preparation and Characterization

The synthetic routes of TT‐In and its precursor TT‐DHIn are depicted in Scheme S1 (Supporting Information). In the preliminary step, the reaction of 5‐(4‐(diphenylamino)phenyl)thiophene‐2‐carbaldehyde and 1‐ethyl‐2,3,3‐trimethyl‐3*H*‐indolium iodide generated the compound TT‐In in 88% yield, which was further reduced using sodium borohydride to afford TT‐DHIn with the yield of 68%. The structures of TT‐In and TT‐DHIn were well characterized by nuclear magnetic resonance (NMR) and high‐resolution mass spectrometry (HRMS)  (Figures S1–S4, Supporting Information).

**Figure 1:**
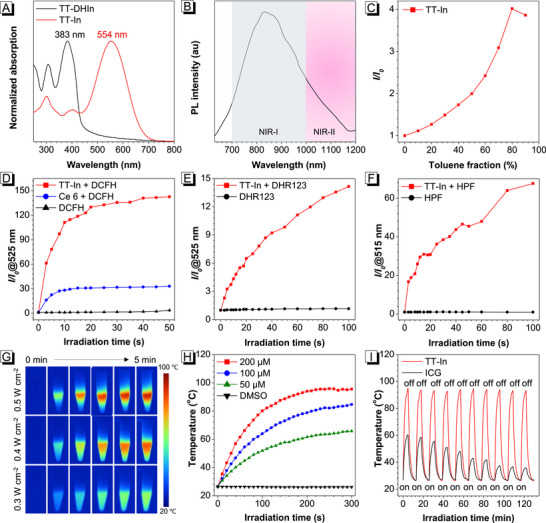
A) Normalized UV–vis absorption spectra of TT‐DHIn (10 µm) and TT‐In (10 µm) in a DMSO/water mixture (*f*_w_ = 99%). B) PL spectra of TT‐In (10 µm) in DMSO. C) The plots of relative maximum emission intensity (*I/I_0_*) of TT‐In in DMSO/toluene mixtures with increasing toluene fractions. D) The plots of relative PL intensity (*I/I_0_*) of DCFH containing TT‐In (2 µm) or Ce6 (2 µm) during 660 nm laser exposure (0.5 W cm^−2^). The plots of relative PL intensity (*I/I_0_*) of E) dihydrorhodamine 123 (DHR123) and F) hydroxyphenyl fluorescein (HPF) containing TT‐In (2 µm) under identical irradiation. G) Photothermal images of TT‐In (200 µm) in DMSO solution under 660 nm laser at different intensities. H) Photothermal conversion behavior of different concentrations of TT‐In in DMSO solution under laser irradiation (0.5 W cm^−2^). I) Anti‐photobleaching property of TT‐In (200 µm) and ICG (200 µm).

The photophysical properties of TT‐DHIn and TT‐In were then studied. As shown in **Figure** **1**A, the main absorption peaks at 383 nm for TT‐DHIn and 554 nm for TT‐In in co‐solvent of DMSO/water (*f*_w_ = 99%) were clearly observed, respectively. Compared with TT‐DHIn, the maximum absorption wavelength of TT‐In was red‐shifted by 171 nm, which was highly favorable for monitoring the transformation of TT‐DHIn into the corresponding counterpart TT‐In via oxidative dehydrogenation. The TT‐In in DMSO solution showed emission tails into the NIR‐II window (Figure 1B), which could be attributed to the high electron donor‐acceptor (D‐A) strength. Moreover, TT‐In permitted NIR II fluorescence signal with a penetration depth of ≈10 mm (Figure S5, Supporting Information), which is twofold deeper than the commercial NIR‐II indocyanine green (ICG). Interestingly, TT‐In always showed a higher SBR ratio at the same penetration depth compared to ICG. With raising the poor solution (toluene) fraction (*f*_T_) from 0 to 90%, a gradually increasing emission intensity of TT‐In was observed, and the emission intensity of TT‐In in the aggregate state was ≈3.9‐fold in DMSO/toluene mixture (*f*_T_ = 90%) compared to that in DMSO solution (Figure 1C; Figures S6 and S7, Supporting Information), conforming its typical AIE behaviors.

To investigate the photodynamic activity of TT‐In under 660 nm laser irradiation, 2,7‐dichlorodihydrofluorescein (DCFH) was used for evaluating the ROS generation ability.^[^ ^54^ ^]^ As shown in Figure 1D and Figure S8 (Supporting Information), the fluorescence intensity of DCFH in the presence of TT‐In was boosted for 143‐fold at 525 nm after 50 s exposure to 660 nm laser irradiation, which exhibited the highest ROS generation efficiency than that of Ce6 (33‐fold) and DCFH (twofold). Therefore, the PDT efficiency of AIE photosensitizer TT‐In was far superior to conventional photosensitizer Ce6. The high photodynamic efficiency of TT‐In could result from the small singlet–triplet energy gaps (Δ*E*_ST_) (Figure S9, Supporting Information). Moreover, it was observed that TT‐In could effectively generate superoxide anion radical (O_2_^•−^) and hydroxyl radical (•OH) upon 660 nm laser irradiation, suggesting its predominant type I ROS activity (Figure 1E,F; Figures S10 and S11, Supporting Information). Compared with 9,10‐anthracenediyl‐bis(methylene) dimalonic acid (ABDA) alone, a gradually decreasing absorption intensity of ABDA at 378 nm was found in the presence of TT‐In (Figure S12, Supporting Information), indicating the type II ROS generation upon irradiation. Furthermore, the photothermal conversion behavior of TT‐In was further evaluated upon 660 nm laser irradiation. TT‐In exhibited laser intensity‐ and concentration‐dependent temperature elevations (Figure 1G,H; Figures S13 and S14, Supporting Information), as well as a high photothermal conversion efficiency of 67.39% (Figure S15, Supporting Information). In addition, the temperature of TT‐In (200 µm) in DMSO solution reached 95.5 °C upon 660 nm laser irradiation (0.5 W cm^−2^) for 300 s, implying the excellent photothermal behavior. Compared with commercial Indocyanine green (ICG), the TT‐In displayed excellent photothermal stability. As shown in Figure 1I, after ten cycles of the laser irradiation/cooling process, the maximum temperature of TT‐In remained basically unchanged, while the maximum temperature of ICG decreased significantly. Furthermore, TT‐In also exhibited excellent anti‐photobleaching performance (Figure S16, Supporting Information).

### 2.2 Activatable Performance of TT‐DHIn

**Figure 2:**
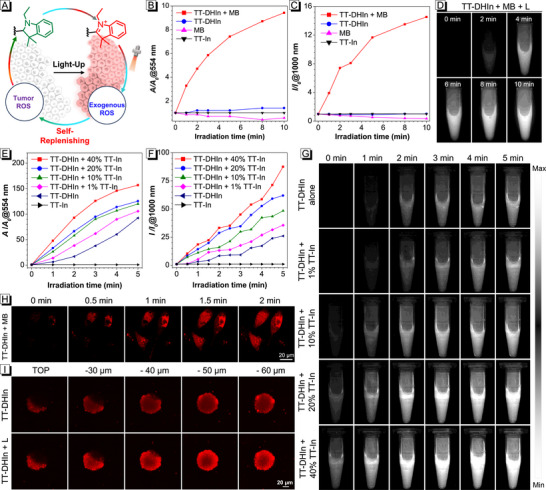
A) Schematic illustration of ROS‐ and light‐cascade activation of TT‐DHIn. Plots of relative B) absorption intensity (*A/A_0_*) at 554 nm and C) PL intensity (*I/I_0_*) at 1000 nm of MB, TT‐In, TT‐DHIn and TT‐DHIn + MB in co‐solvent of DMSO/water under 660 nm laser irradiation (0.2 W cm^−2^) for 0–10 min. D) The NIR‐II fluorescence images of TT‐DHIn + MB under 660 nm laser irradiation (0.2 W cm^−2^) for 0–10 min. Plots of relative E) absorption intensity (*A/A_0_*) at 554 nm and F) PL intensity (*I/I_0_*) at 1000 nm of TT‐DHIn containing different concentrations of TT‐In in co‐solvent of DMSO/water under 660 nm laser irradiation (0.5 W cm^−2^) for 0–5 min. G) The NIR‐II fluorescence images of TT‐DHIn containing different concentrations of TT‐In under 660 nm laser irradiation (0.5 W cm^−2^) for 0–5 min. H) Confocal laser scanning microscopy (CLSM) images of MDA‐MB‐231 cells co‐treated with TT‐DHIn and MB under 638 nm laser irradiation (40% power). I) CLSM images of MDA‐MB‐231 multi‐cell tumor spheroid treated with TT‐DHIn before or after 638 nm laser irradiation (40% power).

The ROS‐ and light‐activatable performance of TT‐DHIn was evaluated (**Figure** **2**). To verify the ROS‐activatable transformation of TT‐DHIn into TT‐In, methylene blue (MB) was employed as an exogenous ROS inducer to generate both Type I and Type II ROS (Figures S17–S19, Supporting Information).^[^ ^55^ ^]^ After 10 min exposure to 660 nm laser irradiation (0.2 W cm^−2^), the TT‐DHIn + MB group exhibited a 9.4‐fold increase in absorption intensity at 554 nm and a 14.5‐fold enhancement in PL intensity at 1000 nm (Figure 2B,C), which could be attributed to the ROS‐activatable transformation of TT‐DHIn into corresponding TT‐In through oxidative dehydrogenation. In contrast, TT‐DHIn alone showed no significant change in both absorption and PL intensities, and gradually decreased absorption and PL intensities for MB were exhibited, mainly due to its poor photostability (Figure S20, Supporting Information).^[^ ^56^ ^]^ Meanwhile, the NIR‐II fluorescence images further verified the ROS‐activatable ability of TT‐DHIn (Figure 2D; Figure S21, Supporting Information). Subsequently, we added different proportions of TT‐In into the TT‐DHIn system to mimic the partial activation of TT‐DHIn in vivo, followed by systematically investigating the photoactivable performance. With the addition of TT‐In fraction from 0 to 40%, the absorption intensity at 554 nm and PL intensity at 1000 nm of TT‐DHIn were amplified by 157‐fold and 87.5‐fold upon 660 nm laser irradiation (0.5 W cm^−2^) (Figure 2E,F), respectively, which could be ascribed to the efficient ROS generation by TT‐In under photo‐irradiation. Moreover, the photoactivatable transformation of TT‐DHIn into TT‐In was further verified by NIR‐II fluorescence images, ^1^H NMR, and  high‐performance liquid chromatography (HPLC) (Figure 2G; Figures S22 and S23, Supporting Information). These results collectively demonstrate that the photoactivation efficiency of TT‐DHIn upon 660 nm laser irradiation exhibits a positive correlation with the concentration of TT‐In. Benefiting from its absorption at 660 nm, TT‐DHIn alone also showed a gradual increase in absorption and PL intensities through prolonging the irradiation time, which could be caused by the transformation of TT‐DHIn into TT‐In via photo‐oxidative dehydrogenation reaction.

With MDA‐MB‐231 cells and MDA‐MB‐231 tumor spheroid as a model, the ROS‐ and light‐cascade activatable performance in vitro of TT‐DHIn is systematically investigated. After co‐incubation with TT‐DHIn and MB, the intracellular fluorescence signal of MDA‐MB‐231 cells gradually increased upon irradiation, whereas that of TT‐DHIn or MB alone exhibited no significant changes (Figure 2H; Figure S24, Supporting Information). This phenomenon could be attributed to the ROS generated by MB under irradiation, which induced TT‐DHIn to transform into TT‐In via oxidative dehydrogenation. To further investigate the photoactivatable performance of TT‐DHIn under partial activation conditions, a 3D MDA‐MB‐231 tumor spheroid model was constructed. As presented in Figure 2I, the 3D tumor spheroid treated with TT‐DHIn was lit up, which could be ascribed to the effective transformation from TT‐DHIn into TT‐In under the activation of ROS in the multi‐cell tumor spheroid microenvironment. After light irradiation for 5 min, its fluorescence intensity was markedly intensified, due to ROS generated by TT‐In under photoactivation to self‐replenish tumor cells, enabling the cyclic activation of TT‐DHIn.

**Figure 3:**
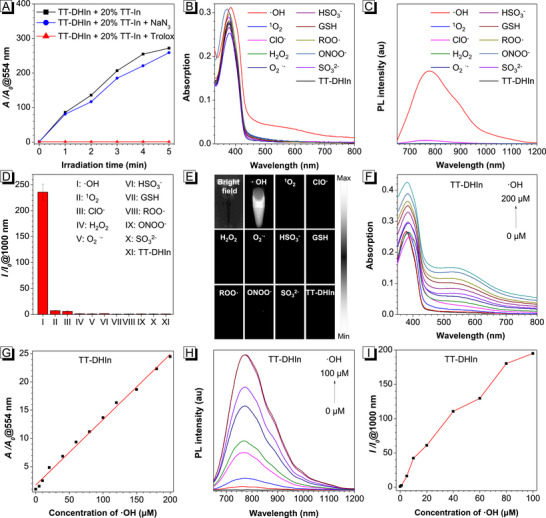
A) Plots of relative absorption intensity (*A/A_0_*) at 554 nm of TT‐DHIn containing 20% TT‐In upon addition of NaN_3_ or Trolox under 660 nm laser irradiation (0.5 W cm^−2^) for 0–5 min, respectively. B) UV–vis absorption spectra, C) PL spectra, D) relative PL intensity (*I/I_0_*) at 1000 nm, and E) NIR‐II fluorescence images of TT‐DHIn treated with various ROS or RNS. F) UV–vis absorption spectra, G) plots of relative absorption (*A/A_0_*) at 554 nm, H) PL spectra, and I) relative PL intensity (*I/I_0_*) at 1000 nm of TT‐DHIn treated with different concentrations of ·OH.

The ROS‐ and light‐activatable mechanism of TT‐DHIn was further systematically investigated. Firstly, verifying whether TT‐DHIn was activated by type I or type II ROS, various ROS‐quenching agents were added into the TT‐DHIn system containing 20%TT‐In. As shown in **Figure** **3**A, after the free radical‐quenching agent (Trolox)^[^ ^57^ ^]^ was added to the TT‐DHIn/TT‐In mixture, the absorption intensity of TT‐DHIn at 554 nm exhibited narrow change upon 660 nm laser irradiation (0.5 W cm^−2^). In contrast, the absorption intensity of TT‐DHIn at 554 nm exhibited similar enhancement in the absence or presence of singlet oxygen‐quenching agent (NaN_3_),^[^ ^58^ ^]^ respectively. These results suggest that TT‐DHIn primarily undergoes oxidative dehydrogenation to transform into TT‐In through the free radical‐activatable pathway. Subsequently, TT‐DHIn was treated with different types of ROS and reactive nitrogen species (RNS) to verify its specific activation performance. Compared with other types of ROS and RNS, the absorption intensity at 554 nm, PL intensity at 1000 nm, and NIR‐II fluorescence signal of TT‐DHIn were significantly enhanced after treatment with hydroxyl radical (·OH) (Figure 3B–E), suggesting the specifically activatable ability toward ·OH. Simultaneously, the absorption and PL intensities of TT‐DHIn also showed excellent ·OH concentration dependence (Figure 3F–I). Therefore, a potential activation mechanism is proposed (Figure S25, Supporting Information): TT‐DHIn can be transformed into TT‐In via oxidative dehydrogenation mediated by ·OH. Then, TT‐In transitions from the ground state to the excited triplet state upon near‐infrared (NIR) light irradiation. Subsequently, ·OH is generated via electron transfer during the transition from the excited singlet state to the excited triplet state, which can further activate TT‐DHIn to transform into TT‐In, achieving the cascade activation characteristics of both ROS and NIR light. In addition, after treatment with HPF (50 µm) for 3 h at 37°C under dark condition, a strong green fluorescence signal was detected for the MDA‐MB‐231 tumor spheres (Figure S26, Supporting Information), suggesting that abundant ·OH in the TME can effectively activate TT‐DHIn transform into the TT‐In (Figure 2I).

### 2.3 Intracellular Distribution and Photodynamic Activity in Vitro

**Figure 4:**
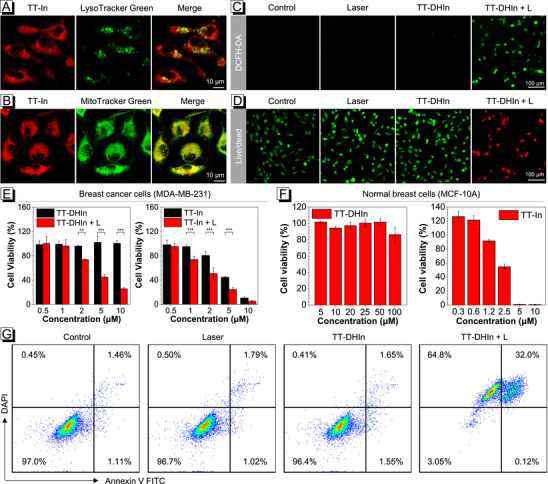
A,B) The intracellular distribution of TT‐In in MDA‐MB‐231 cells. C) Laser‐induced (660 nm, 0.5 W cm^−2^) ROS generation in MDA‐MB‐231 cells under different administrations. D) Live and dead staining assay of MDA‐MB‐231 cells after various treatments, with green and red fluorescence indicating live and dead cells, respectively. Dose‐dependent viability of E) MDA‐MB‐231 cancer cells and F) MCF‐10A normal cells treated with TT‐DHIn or TT‐In, with or without 660 nm laser irradiation (0.5 W cm^−2^). G) Apoptosis analysis of MDA‐MB‐231 cells via flow cytometry across different intervention groups. Values represent means ± SD (n = 3), bars with different characters are statistically different at ^*^ *p* <0.05, ^**^ *p* <0.01, ^***^ *p* <0.001 level versus control.

The intracellular distribution of TT‐In was further investigated in the MDA‐MB‐231 cells by colocalization assay. As shown in **Figure** **4**A,B, a higher Pearson's correlation coefficient (0.93) for TT‐In/MitoTracker was observed compared with that of TT‐In/LysoTracker (0.61), suggesting its excellent mitochondrial targeting ability. The intracellular ROS generation of TT‐DHIn and TT‐In upon 660 nm laser irradiation was evaluated using DCFH‐DA^[^ ^59^ ^]^ as an indicator, which showed a strong green fluorescence signal in MDA‐MB‐231 cells (Figure 4C; Figure S27, Supporting Information). Meanwhile, the corresponding tendency was also verified by flow cytometric quantification of ROS‐positive cells (Figure S28, Supporting Information), suggesting its excellent ROS generation ability. Further investigation revealed that TT‐In and TT‐DHIn could effectively reduce mitochondrial the membrane potential of MDA‐MB‐231 cells upon irradiation (Figure S29, Supporting Information). Subsequently, the live/dead assay demonstrated that TT‐DHIn combined with laser irradiation could effectively kill in vitro cancer cells compared to control groups (Figure 4D).

TT‐DHIn showed excellent stability in the DMEM system (Figure S30, Supporting Information). Subsequently, the in vitro cytotoxicity of TT‐DHIn and TT‐In was further evaluated with MDA‐MB‐231 cells and normal MCF‐10A cells. As illustrated in Figure 4E, the cell viability of MDA‐MB‐231 cells treated with TT‐DHIn (10 µm) decreased to 26% under 660 nm laser irradiation, yet that of MDA‐MB‐231 cells under dark conditions exhibited much higher cell viability of nearly 100%. While TT‐In displayed significant cytotoxicity against MDA‐MB‐231 cells, an extremely low cell viability (0.8%) against normal MCF‐10A cells was observed at a concentration of 5 µm (Figure 4F). The excellent biosafety of TT‐DHIn was verified in normal MCF‐10A breast cells even at a high concentration of 100 µm. These results indicated that TT‐DHIn could not only achieve superior phototherapeutic efficacy against cancer cells but also significantly reduce off‐targeted drug toxicity. Finally, the cell death mechanism of MDA‐MB‐231 cells under phototherapy was assessed by the Annexin V‐FITC/DAPI assay. As shown in Figure 4G, the apoptosis rate of MDA‐MB‐231 cells treated with both TT‐DHIn and laser irradiation was 32.12%, while a low apoptosis rates were observed for control, laser, and TT‐DHIn under dark conditions. Meanwhile, the TT‐In + L group also exhibited a high apoptosis rate (Figure S31, Supporting Information). These results suggest the excellent phototherapeutic efficacy of TT‐DHIn and TT‐In via the apoptosis mechanisms.

### 2.4 In Vivo Phototherapy and Biosafety Evaluation

**Figure 5:**
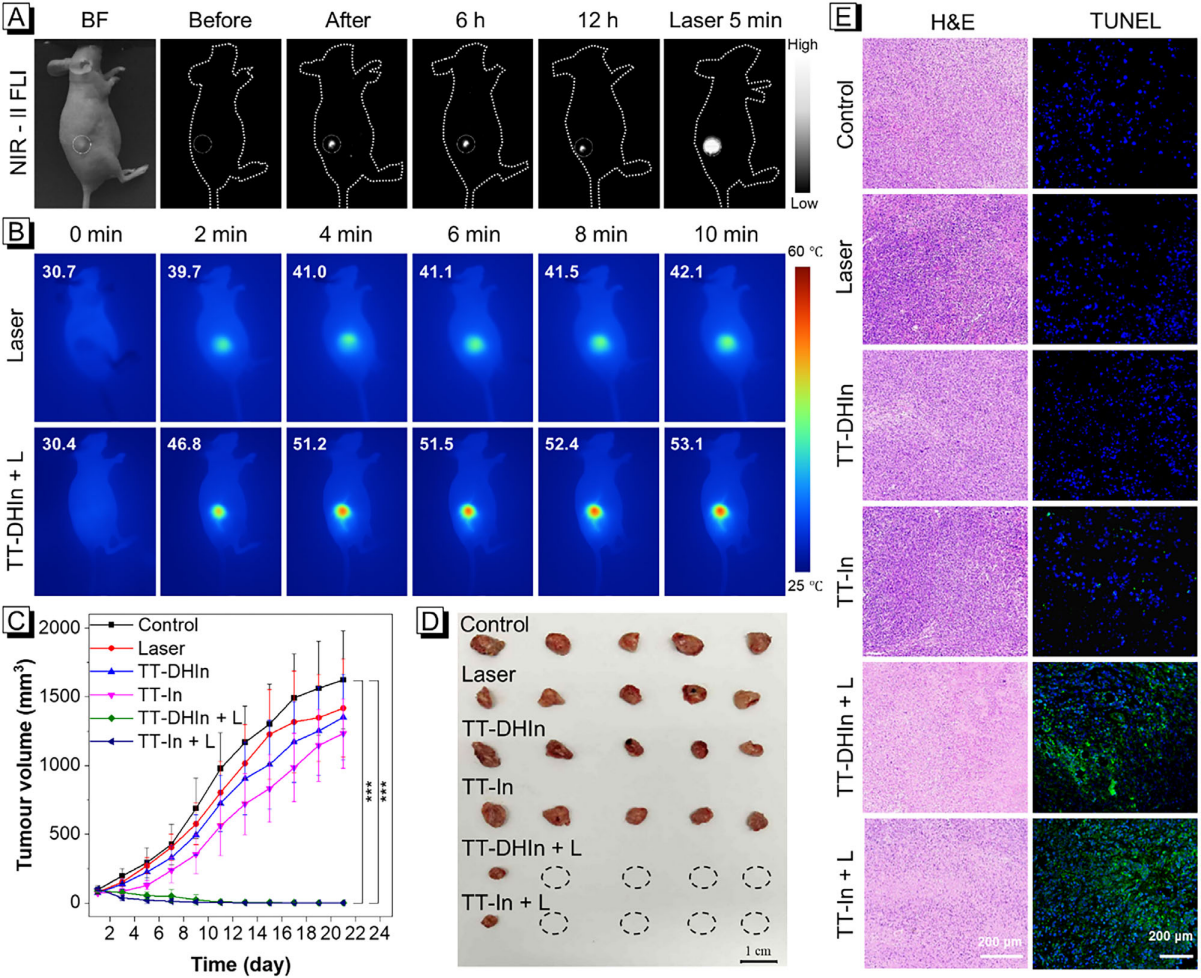
A) In vivo ROS/light‐activatable NIR‐II fluorescence imaging in MDA‐MB‐231 tumor models post‐TT‐DHIn treatment. B) Photothermal imaging of tumors following saline or TT‐DHIn injection under 660 nm laser irradiation (0–10 min). C) Tumor growth curves over a 21‐day period across the various treatment groups. D) The photographs of tumors harvested from different treatment groups at day 21. E) H&E and TUNEL staining of tumor sections from mice after various treatments. Values represent means ± SD (n = 5), bars with different characters are statistically different at ^*^ *p* <0.05, ^**^ *p* <0.01, ^***^ *p* <0.001 level versus control.

To further investigate the ROS‐ and light‐cascade activatable properties of TT‐DHIn in vivo, the nude mice bearing MDA‐MB‐231 tumors were established. As shown in **Figure** **5**A, light‐up NIR‐II fluorescence signals with a high SBR of 11.5 were observed at the tumor location post‐injection of TT‐DHIn, which could be attributed to the rapidly transformation from TT‐DHIn to TT‐In via oxidative dehydrogenation under tumor microenvironmental ROS activation. In contrast, the ICG only showed a low SBR of 1.8 at the tumor site (Figure S32, Supporting Information). These results suggest that ROS‐ and light‐cascade activatable TT‐DHIn is particularly suitable for tumor imaging with high SBR. Subsequently, the NIR‐II fluorescence signal of tumor site could be used as a photoactivatable “guiding radar”, followed by selective irradiation of tumor with a 660 nm laser for 5 min, utilizing the high spatiotemporal controllability of light. The NIR‐II fluorescence intensity at the tumor site was significantly enhanced, which was attributed to the self‐replenishment of ROS in the tumor region via ROS generated by intratumor TT‐In upon 660 nm laser irradiation. Moreover, residual TT‐DHIn in tumor site could be also cyclically transformed into TT‐In, amplifying its phototherapeutic activity. Notably, the tumor temperature of mice treated with TT‐DHIn reached 53.1 °C under 660 nm laser irradiation for 10 min (Figure 5B), demonstrating effective hyperthermia for tumor phototherapy. In contrast, minimal temperature fluctuation was recorded in the saline group. Moreover, we systematically evaluated the in vivo antitumor performance of TT‐DHIn. As shown in Figure 5C, the tumors of MDA‐MB‐231‐bearing nude mice treated with TT‐DHIn and TT‐In were efficiently eradicated upon 660 nm laser irradiation compared to those of other treatment conditions. Meanwhile, the tumor tissues of the different treatment groups were dissected post 21‐day therapy, and the photographs, weight, hematoxylin and eosin (H&E) and TUNEL staining of ex vivo tumor tissues collectively affirmed the significant tumor phototherapy for TT‐DHIn + L group and TT‐In + L group (Figures 5D,E; Figure S33, Supporting Information). We also quantitatively analyzed the area of the apoptotic positive signal regions based on TUNEL staining of tissue sections. As shown in Figure S34 (Supporting Information), the tunnel positive signals in the TT‐DHIn + L group and the TT‐In + L group were significantly increased compared to other groups, which were attributed to the effective activation of the phototherapeutic activity of TT‐DHIn under both ROS and 660 nm laser irradiation. In addition, the body weight of MDA‐MB‐231‐bearing nude mice treated with TT‐DHIn and TT‐In exhibited negligible changes during 21‐day treatment process (Figure S35, Supporting Information). Furthermore, hemolysis assay, H&E stained images of main organs and blood biochemistry indices analysis further verified excellent biosafety and low systematic toxicity of this ROS‐ and light‐cascade activatable NIR‐II AIE probe toward to normal tissue organs (Figures S36–S38, Supporting Information).

## 3 Conclusion

In summary, we developed a versatile ROS‐ and NIR light‐cascade activatable NIR‐II AIE theranostic agent TT‐DHIn, which exhibits specific ·OH responsiveness, high photoactivatable efficiency, and excellent biosafety. Upon activation, TT‐DHIn shows NIR‐II emission, strong ·OH generation capability, and efficient photothermal conversion. Using ROS‐activatable light‐up NIR‐II fluorescence imaging, we achieved clear visualization of in vitro multicellular tumor spheroids and in vivo subcutaneous tumors with an outstanding signal‐to‐background ratio. This imaging capability enabled precise photoactivation guidance, significantly enhancing tumor PDT/PTT efficacy through the cyclic activation of TT‐DHIn. The dual‐activatable cascade amplification strategy innovatively integrates tumor biomarker (ROS) responsiveness with a photoactivation mechanism, offering great promise for imaging‐guided precise tumor therapy, as well as further boosting the state‐of‐the‐art advance of tumor phototheranostics in clinical trials.

## 4 Experimental Section

### Synthesis and Characterization of TT‐In

Dry ethanol (10 mL) was added to 5‐(4‐(diphenylamino)phenyl)thiophene‐2‐carbaldehyde^[^ ^60^ ^]^ (213 mg, 0.6 mmol) and 1‐ethyl‐2,3,3‐trimethyl‐3H‐indol‐1‐ium iodide^[^ ^61^ ^]^ (158 mg, 0.5 mmol) under nitrogen. Following 10 h of reflux under nitrogen and cooling, the mixture was concentrated under reduced pressure. The residue was dissolved in acetone, combined with saturated KPF₆ solution (3 mL), and stirred for 6 h. Subsequent evaporation of the solvent yielded a precipitate, which was isolated by vacuum filtration, washed with water, and dried. Purification by column chromatography (silica, DCM/MeOH 20:1) yielded TT‐In (287.5 mg, 88%). ^1^H NMR (500 MHz, DMSO‐*d*_6_) δ 8.68 (d, *J* = 15.7 Hz, 1H), 8.23 (d, *J* = 4.1 Hz, 1H), 7.89–7.83 (m, 2H), 7.76–7.70 (m, 3H), 7.63–7.55 (m, 2H), 7.38 (t, *J* = 7.9 Hz, 4H), 7.24–7.11 (m, 7H), 7.00–6.96 (m, 2H), 4.62 (q, *J* = 7.2 Hz, 2H), 1.78 (s, 6H), 1.43 (t, *J* = 7.2 Hz, 3H). ^13^C NMR (126 MHz, DMSO‐*d*_6_) δ 179.81, 154.54, 148.97, 146.22, 146.09, 143.52, 140.53, 140.35, 138.24, 129.86, 129.06, 128.74, 127.47, 125.41, 125.27, 124.43, 123.03, 121.30, 114.49, 108.80, 51.72, 41.51, 25.77, 13.53. HRMS (ESI): m/z [M] + calcd for C_36_H_33_N_2_S: 525.2359, found: 525.2355

### Synthesis and Characterization of TT‐DHIn

TT‐In (196 mg, 0.3 mmol) was dissolved in methanol (20 mL) at 0 °C. A solution of sodium borohydride (13 mg, 0.36 mmol) in ethanol was then added dropwise with vigorous stirring. Following a 10‐min reaction, the mixture was evaporated under reduced pressure. The residue was slowly quenched with ice water (10 mL) and extracted with dichloromethane. Subsequent concentration of the organic phase and purification by column chromatography (silica, PE/DCM = 3:1) yielded TT‐DHIn (107 mg, 68%). ^1^H NMR (500 MHz, DMSO‐*d*_6_) δ 7.56 (dd, *J* = 8.8, 2.3 Hz, 2H), 7.35–7.30 (m, 5H), 7.12–7.00 (m, 9H), 6.98–6.94 (m, 2H), 6.90 (dd, *J* = 15.6, 1.7 Hz, 1H), 6.62 (t, *J* = 7.3 Hz, 1H), 6.54–6.49 (m, 1H), 5.99 (dd, *J* = 15.7, 9.1 Hz, 1H), 3.70 (d, *J* = 9.0 Hz, 1H), 3.31–3.27 (m, 1H), 3.03 (dq, *J* = 14.1, 7.0 Hz, 1H), 1.27 (d, *J* = 1.2 Hz, 3H), 1.02–0.97 (m, 6H). ^13^C NMR (126 MHz, DMSO‐*d*_6_) δ 149.60, 147.34, 147.27, 142.45, 140.34, 138.89, 130.11, 128.42, 127.90, 127.82, 127.58, 126.99, 126.86, 124.80, 123.93, 123.64, 123.38, 122.15, 117.91, 107.74, 75.92, 44.43, 26.25, 24.58, 10.33. HRMS (ESI): m/z [M] + calcd for C_36_H_34_N_2_S: 526.2443; found: 526.2418.

### ROS‐Activatable Cell Imaging

Following overnight adhesion in glass‐bottom dishes (3×10⁴ cells/dish), MDA‐MB‐231 cells were PBS‐washed and treated with 10 µm TT‐DHIn for 8 h. Fresh DMEM containing 50 µm MB was then added for a subsequent 5 h incubation. Prior to imaging, cells were thoroughly rinsed with PBS and replenished with fresh medium. Confocal images (red channel: λ_ex_ = 552 nm, λ_em_ = 650–793 nm) were captured during 0–2 min of 638 nm laser irradiation.

### Cell Apoptosis Analysis

Following overnight culture of MDA‐MB‐231 cells in 6‐well plates (2 × 10⁵ cells/well) at 37 °C, the cultures were washed with PBS and then subjected to an 8 h treatment with TT‐DHIn or TT‐In (10 µm). The cells were then irradiated for 10 min using a 660 nm laser (0.5 W cm^−2^). After an additional 18 h of incubation, the cells were collected, stained with Annexin V‐FITC/DAPI for 5 min, and analyzed for apoptosis via flow cytometry.

### In Vivo Therapy Experiment

All animal procedures were approved by the Animal Center of Guangdong Medical University (GDMU‐2023‐000096). Nude mice bearing MDA‐MB‐231 xenografts (3 × 10⁶ cells) were randomly grouped (n = 5) when tumor volumes approached 100 mm^3^. The groups included: Control (0.9% NaCl), Laser only, TT‐DHIn (2.5 mg kg^−1^), TT‐DHIn + Laser, TT‐In (2.5 mg kg^−1^), and TT‐In + Laser. Treatments were delivered intratumorally. After 12 h, the tumor sites in laser groups were irradiated (660 nm, 0.5 W cm^−2^, 10 min). Tumor volume (V = a × b^2^ / 2) was recorded every other day for 21 days. After the treatment period, all animals were sacrificed, and organs and blood were collected for H&E staining, TUNEL assay, and biochemical analysis.

### Statistical Analysis

Each experiment was performed in triplicate, with results expressed as mean ± SD. Statistical analysis was carried out using GraphPad Prism 8 software. Intergroup differences were evaluated by a two‐tailed Student's t‐test, and statistical significance was set at ^*^p <0.05, ^**^p <0.01, and ^***^p <0.001. All figures were plotted with Origin 8.5 software.

## Conflict of Interest

The authors declare no conflict of interest.

## Supporting information



Supporting Information

## Data Availability

The data that support the findings of this study are available in the supplementary material of this article.
