# Supplementary material for: From Spark to Flame: ROS‐ and Light‐Cascade Activatable NIR‐II AIE Probe for Precise Tumor Imaging and Self‐Amplifying Phototherapy
Source: Adv Sci (Weinh). 2025 Nov 2;13(3):e14789. doi: 10.1002/advs.202514789 (PMC12806408; doi:10.1002/advs.202514789)
Supplement: Supplementary file 1 — Supporting Information [file ADVS-13-e14789-s001.docx]

Supporting Information

From Spark to Flame: ROS- and Light-Cascade Activatable NIR-II AIE Probe for Precise Tumor Imaging and Self-Amplifying Phototherapy

Xiaohui Chen,^#^* Yuanyuan You,^#^ Songling Lin,^#^ Chengwei Tang, Jun Zhu, Qiongwen Liang, Dan Rao, Jiali Deng, Yuxun Ding, Dingyuan Yan, Wenman Li, Xiaohui Chen,* Dong Wang,* and Ben Zhong Tang*

Table of Contents

| **Experimental Procedures** | 4 |
| --- | --- |
| **Materials** | 4 |
| **Instruments** | 4 |
| **ROS generation** | 4 |
| **^1^O_2_ detection** | 4 |
| **O_2_^•−^ detection** | 5 |
| **•OH detection** | 5 |
| **Photothermal conversion measurement** | 5 |
| **Cell culture** | 5 |
| **Colocalization experiment** | 5 |
| **Cell viability measurement** | 6 |
| **Scheme S1.** The synthetic routes of TT-In and TT-DHIn | 7 |
| **Figure S1.** ^1^H and ^13^C NMR spectra of TT-In in DMSO-*d_6_* | 8 |
| **Figure S2.** ^1^H and ^13^C NMR spectra of TT-DHIn in DMSO-*d_6_* | 9 |
| **Figure S3.** The HRMS spectra of TT-In | 10 |
| **Figure S4.** The HRMS spectra of TT-DHIn | 11 |
| **Figure S5.** NIR-II fluorescence images and (B) corresponding SBRs of TT-In and ICG in aqueous solution filled in capillaries and immersed in 1% Intralipid with varying depths | 12 |
| **Figure S6.** PL spectra of TT-In versus the solvent composition changes of DMSO/toluene mixture | 13 |
| **Figure S7.** The size distribution histogram of TT-In in toluene/DMSO (*f*_T_ = 90%) solution | 14 |
| **Figure S8.** The PL spectra of DCFH, Ce6 and TT-In + DCFH under 660 nm laser irradiation for 0–50 s | 15 |
| **Figure S9.** HOMO–LUMO electronic cloud distributions and energy level difference between singlet and triplet state of TT-DHIn and TT-In by DFT calculations | 16 |
| **Figure S10.** The PL spectra of DHR123 and TT-In + DHR123 under 660 nm laser irradiation for 0–100 s | 17 |
| **Figure S11.** The PL spectra of HPF and TT-In + HPF under 660 nm laser irradiation for 0–100 s | 18 |
| **Figure S12.** The absorption spectra and relative absorption intensity of ABDA in the absence and presence of TT-In under 660 nm laser irradiation for 0–100 s | 19 |
| **Figure S13.** Photothermal conversion behavior of TT-In under 660 nm laser irradiation at different laser intensity | 20 |
| **Figure S14.** Photothermal conversion behavior of TT-In at different concentrations under 660 nm laser irradiation | 21 |
| **Figure S15.** Photothermal conversion efficiency of TT-In under 660 nm laser irradiation  **Figure S16.** The absorption spectra and plots of relative absorption intensity (*A/A_0_*) at 554 nm of TT-In and ICG upon continuous 660 nm laser irradiation for 20 min | 22  23 |
| **Figure S17.** Relative absorption intensity of ABDA in the absence and presence of MB under 660 nm laser irradiation for 0–90 s | 24 |
| **Figure S18.** The PL spectra and relative PL intensity of DHR123 in the absence and presence of MB under 660 nm laser irradiation for 0–70 s | 25 |
| **Figure S19.** The PL spectra and relative PL intensity of HPF in the absence and presence of MB under 660 nm laser irradiation for 0–100 s | 26 |
| **Figure S20.** The absorption spectra and relative absorption intensity of MB under 660 nm laser irradiation at different laser intensity for 0–10 min | 27 |
| **Figure S21.** The NIR-II fluorescence imaging of TT-DHIn and MB under 660 nm laser irradiation for 0–10 min | 28 |
| **Figure S22.** The ^1^H NMR stacking spectra of TT-DHIn containing 10%TT-In in THF-*d*_8_ under 660 nm laser irradiation for 0–60 min | 29 |
| **Figure S23.** The HPLC stacking spectra of TT-DHIn containing 10%TT-In in MeCN under 660 nm laser irradiation for 0–90 min | 30 |
| **Figure S24.** CLSM images of MDA-MB-231 cells treated with TT-DHIn and MB under 638 nm laser irradiation  **Figure S25.** The speculated mechanism of transformation from TT-DHIn into TT-In via oxidative dehydrogenation | 31  32 |
| **Figure S26.** CLSM images of MDA-MB-231 multi-cell tumor spheroid treated with HPF | 33 |
| **Figure S27.** Fluorescence images of intracellular ROS accumulation in MDA-MB-231 cells treated with TT-In with or without irradiation  **Figure S28.** Flow cytometric analysis of MDA-MB-231 cells co-stained with TT-DHIn and TT-In with DCFH-DA under 660 nm laser irradiation for 0–180 s  **Figure S29.** Flow cytometry analysis of mitochondrial membrane potential dissipation co-stained with TT-DHIn and TT-In using JC-1 as an indicator  **Figure S30.** The absorption intensities at 383 nm of TT-DHIn in DMEM system over a 7-day period  **Figure S31.** Flow cytometric analysis of MDA-MB-231 cells after different interventions | 34  35  36  37  38 |
| **Figure S32.** The NIR-II fluorescence images of nude mice bearing the MDA-MB-231 tumor after treatment with ICG | 39 |
| **Figure S33.** The average tumor weight of nude mice after various treatments for 21 d | 40 |
| **Figure S34.** Fluorescence statistical graph of positive apoptotic signals in TUNEL staining | 41 |
| **Figure S35.** The body weight of nude mice during treatment process | 42 |
| **Figure S36.** Hemolytic effect of TT-DHIn and TT-In on mice RBCs | 43 |
| **Figure S37.** H&E staining of the major organs sections from MDA-MB-231-bearing nude mice after various treatments for 21 d | 44 |
| **Figure S38.** Blood biochemistry indices (BUN, AST, ALT, and TP) of after various treatments for 21 d | 45 |
| **References** | 46 |

Experimental Procedures

**Materials**

Sodium borohydride was purchased from Tianjin Damao Chemical Reagent Co., Ltd (Tianjin, China). 2, 7-dichlorodihydrofluorescein diacetate (DCFH-DA), 9,10-anthracenediyl-bis(methylene) dimalonic acid (ABDA), and Dihydrorhodamine 123 (DHR123) were purchased from Sigma-Aldrich. Hydroxyphenyl fluorescein (HPF) was purchased from Mkbio (Shanghai, China). Methylene blue (MB) was purchased from MCE. Annexin V-488/DAPI apoptosis detection kit was purchased from Dojindo. Cell Counting Kit-8 (CCK-8) was purchased from Beyotime Institute of Biotechnology (Haimen, China). MitoTracker Green and LysoTracker Green were purchased from Life Technologies.

**Instruments**

^1^H and ^13^C NMR spectra were measured on a Bruker ARX 500 NMR spectrometer. High-resolution mass spectra (HRMS) were measured on a Xevo G2-XS Q-Tof Mass spectrometer. UV–Vis absorption spectra were recorded on a PerkinElmer Lambda 950. Photoluminescence spectra were measured on a FS 5 fluorescence spectrometer. Confocal lasing scanning microscopic (CLSM) images were obtained on a confocal microscope (Leica, TCS SP8). 660 nm laser was provided by Changchun radium photoelectric technology (Changchun, China). The cell viability analysis was performed using a microplate reader (Biotek Cytation 5).

**ROS generation**

The ROS generation was measured using 2,7-dichlorodihydrofluorescein (DCFH) as an indicator. Firstly, the DCFH-DA (0.5 mL, 1 mM in ethanol) was added to 2 mL of NaOH (0.01 mM), followed by reaction for 30 min under dark condition at room temperature. The mixture was then neutralized with 10 mL PBS to get DCFH (40 μM). The TT-In was added into PBS buffer containing DCFH, followed by irradiation under 660 nm laser (0.5 W cm^–2^) for 0–50 s. The fluorescence signals of DCF were monitored by using a fluorescence spectrometer. [DCFH] = 5 μM, [TT-In] = 2 μM, λ_ex_ = 488 nm, λ_em_ = 500–600 nm.

**^1^O_2_ detection**

The ^1^O_2_ generation was measured using ABDA as an indicator. The TT-In was added into PBS buffer containing ABDA, followed by irradiation under 660 nm laser (0.5 W cm^–2^) for 0–100 s. The absorption spectra of ABDA were monitored by using a UV–Vis absorption spectrometer. [ABDA] = 10 μM, [TT-In] = 2 μM.

**O_2_^•−^ detection**

The O_2_^•−^ generation was measured using DHR123 as an indicator. The TT-In was added into PBS buffer containing DHR123, followed by irradiation under 660 nm laser (0.5 W cm^–2^) for 0–100 s. The fluorescence signals of indicator were monitored by using a fluorescence spectrometer. [DHR123] = 10 μM, [TT-In] = 2 μM, λ_ex_ = 488 nm, λ_em_ = 500–600 nm.

•**OH detection**

The •OH generation was measured using HPF as an indicator. The TT-In was added into PBS buffer containing HPF, followed by irradiation under 660 nm laser (0.5 W cm^–2^) for 0–100 s. The fluorescence signals of indicator were monitored by using a fluorescence spectrometer. [HPF] = 10 μM, [TT-In] = 2 μM, λ_ex_ = 488 nm, λ_em_ = 500–600 nm.

**Photothermal conversion measurement**

TT-In dissolved in DMSO was continuously exposed to a 660 nm laser at varied power density for 300 s. The temperature was recorded by an IR thermography every 10 s and stopped until the temperature reached to a plateau. And pure DMSO solution under the same condition was served as the control. In addition, the photothermal conversion efficiency (η) of TT-In in DMSO solution was calculated according to the literature.^[1]^

**Cell culture**

MDA-MB-231 cells were purchased from ATCC and cultured in DMEM (Dulbecco’s Modified Essential Medium) with 1% penicillin-streptomycin and 10% FBS (fetal bovine serum) at 37 °C in a humidified incubator with 5% CO_2_.

**Colocalization experiment**

The MDA-MB-231 cells were seeded in glass-bottom cell culture dishes (3 × 10^4^ cells/dish). After overnight incubation, the MDA-MB-231 cells were washed with PBS followed by treating with TT-In (2 μM) for 2 h. After being washed three times with PBS, the MDA-MB-231 cells were stained with LysoTracker Green (10 μM) or MitoTracker green (10 μM) for 15 min. The cells were rinsed with PBS and fresh culture media was added for fluorescence imaging. For red channel, λ_ex_ = 552 nm, λ_em_ = 650–793 nm; For green channel, λ_ex_ = 488 nm, λ_em_ = 500–550 nm.

**Cell viability measurement**

Cell viability of MDA-MB-231 cancer cells: The MDA-MB-231 cells were seeded into 96-well plates (5 × 10^3^ cells/well) for 24 h. Subsequently, the cells were rinsed with PBS and further treated with various concentrations of TT-DHIn and TT-In. After incubation for 8 h, the MDA-MB-231 cells treated with TT-DHIn or TT-In were irradiated under 660 nm laser (0.5 W cm^–2^) for 10 min, respectively. For control groups, the MDA-MB-231 cells were kept under dark condition. After that, the cells were further incubated for 18 h, and rinsed with PBS for three times. Finally, the CCK-8 assay was used for measuring cell viability of MDA-MB-231 cells.

Cell viability of MCF-10A normal cells: The MCF-10A cells were seeded into 96-well plates (5 × 10^3^ cells/well) for 24 h. Subsequently, the cells were rinsed with PBS and further treated with various concentrations of TT-DHIn and TT-In. After incubation for 24 h, the MCF-10A cells were rinsed with PBS for three time. Finally, the CCK-8 assay was used for measuring cell viability of MCF-10A cells.

**Scheme S1.** The synthetic routes of TT-In and TT-DHIn.

**Figure S1.** ^1^H and ^13^C NMR spectra of TT-In in DMSO-*d_6_*.

**Figure S2.** ^1^H and ^13^C NMR spectra of TT-DHIn in DMSO-*d_6_*.

**Figure S3.** The HRMS spectrum of TT-In.

**Figure S4.** The HRMS spectrum of TT-DHIn.

**Figure S5.** (A) NIR-II fluorescence images and (B) corresponding SBRs of TT-In and ICG in aqueous solution filled in capillaries and immersed in 1% Intralipid with varying depths. Values represent means ± SD (n = 3).

**Figure S6.** PL spectra of TT-In versus the solvent composition changes of DMSO/toluene mixture.

**Figure S7.** The size distribution histogram of TT-In in toluene/DMSO (*f*_T_ = 90%) solution.

**Figure S8.** The PL spectra of (A) DCFH (B) Ce6 and (C) TT-In+ DCFH under 660 nm laser irradiation (0.5 W cm^–2^) for 0–50 s. [DCFH] = 5 μM, [TT-In] = [Ce6] = 2 μM.

**Figure S9.** HOMO–LUMO electronic cloud distributions and energy level difference between singlet and triplet state of TT-DHIn and TT-In by DFT calculations.

**Figure S10.** The PL spectra of (A) DHR123 and (B) TT-In + DHR123 under 660 nm laser irradiation (0.5 W cm^–2^) for 0–100 s. [DHR123] = 10 μM, [TT-In] = 2 μM.

**Figure S11.** The PL spectra of (A) HPF and (B) TT-In + HPF under 660 nm laser irradiation (0.5 W cm^–2^) for 0–100 s. [HPF] = 10 μM, [TT-In] = 2 μM.

**Figure S12.** (A-B) The absorption spectra and (C) relative absorption intensity of ABDA in the absence and presence of TT-In under 660 nm laser irradiation (0.5 W cm^–2^) for 0–100 s. [ABDA] = 10 μM, [TT-In] = 2 μM.

**Figure S13.** Photothermal conversion behavior of (A) TT-In (200 μM), (B) TT-In (100 μM) and (C) TT-In (50 μM) under 660 nm laser irradiation at different laser intensity.

**Figure S14.** Photothermal conversion behavior of TT-In at different concentrations under 660 nm laser irradiation at (A) 0.4 W cm^–2^ and (B) 0.3 W cm^–2^.

**Figure S15.**  Photothermal conversion efficiency of TT-In (100 μM) under 660 nm laser irradiation (0.5 W cm^–2^).

**Figure S16.** (A-B) The absorption spectra and (C) plots of relative absorption intensity (*A/A_0_*) at 554 nm of TT-In and ICG upon continuous 660 nm laser irradiation (0.5 W cm^–2^) for 20 min. [TT-In] = [ICG] = 10 μM.

**Figure S17.** Relative absorption intensity of ABDA in the absence and presence of MB under 660 nm laser irradiation (0.2 W cm^–2^) for 0–90 s. [ABDA] = 25 μM, [MB] = 5 μM.

**Figure S18.** (A-B) The PL spectra and (C) relative PL intensity of DHR123 in the absence and presence of MB under 660 nm laser irradiation (0.2 W cm^–2^) for 0–70 s. [DHR123] = 25 μM, [MB] = 5 μM.

**Figure S19.** (A-B) The PL spectra and (C) relative PL intensity of HPF in the absence and presence of MB under 660 nm laser irradiation (0.2 cm^–2^) for 0–100 s. [HPF] = 25 μM, [MB] = 5 μM.

**Figure S20.** The absorption spectra and relative absorption intensity of MB under 660 nm laser irradiation at different laser intensity for 0–10 min.

**Figure S21.** The NIR-II fluorescence imaging of TT-DHIn and MB under 660 nm laser irradiation (0.2 W cm^–2^) for 0–10 min, respectively.

**Figure S22.** The ^1^H NMR stacking spectra of TT-DHIn containing 10%TT-In in THF-*d_8_* under 660 nm laser irradiation for 0–60 min.

**Figure S23.** The HPLC stacking spectra of TT-DHIn containing 10%TT-In in MeCN under 660 nm laser irradiation for 0–90 min.

**Figure S24.** CLSM images of MDA-MB-231 cells treated with (A) TT-DHIn and (B) MB under 638 nm laser irradiation (40% power).

**Figure S25.** The speculated mechanism of transformation from TT-DHIn into TT-In via oxidative dehydrogenation.

**Figure S26.** CLSM images of MDA-MB-231 multi-cell tumor spheroid treated with HPF (50 μM).

**Figure S27.** Fluorescence images of intracellular ROS accumulation in MDA-MB-231 cells treated with TT-In under dark or irradiation conditions.

**Figure S28.** Flow cytometric analysis of MDA-MB-231 cells treated with (A-B) TT-DHIn or (C-D) TT-In, respectively, followed by staining with DCFH-DA (10 μM) under 660 nm laser irradiation for 0–180 s. [TT-DHIn] = [TT-In] = 10 μM.

**Figure S29.** Flow cytometry analysis of mitochondrial membrane potential (ΔΨ_m_) dissipation for MDA-MB-231 cells treated with (A) TT-DHIn or (B)TT-In using JC-1 dye as an indicator. [TT-DHIn] = [TT-In] = 10 μM.

**Figure S30.** The absorption intensities at 383 nm of TT-DHIn in DMEM system over a 7-day period. Values represent means ± SD (n = 3).

**Figure S31.** Flow cytometric analysis of MDA-MB-231 cells after different interventions. Values represent means ± SD (n = 3).

**Figure S32.** The NIR-II fluorescence images of nude mice bearing the MDA-MB-231 tumor after treatment with ICG.

**Figure S33.** The average tumor weight of nude mice after various treatments for 21 d. Values represent means ± SD (n = 5), bars with different characters are statistically different at **P* < 0.05, ***P* < 0.01, ****P* < 0.001 level versus control.

**Figure S34.** Fluorescence statistical graph of positive apoptotic signals in TUNEL staining. Values represent means ± SD (n = 3), bars with different characters are statistically different at **P* < 0.05, ***P* < 0.01, ****P* < 0.001 level versus control.

**Figure S35.** The body weight of nude mice during treatment process. Values represent means ± SD (n = 5).

**Figure S36.** Hemolytic effect of (A) TT-DHIn and (B) TT-In on mouse RBCs at the concentrations ranging from 2 to 20 μM, using PBS as the negative control and triton X-100 as the positive control. Values represent means ± SD (n = 3).

**Figure S37.** H&E staining of the major organs’ sections from MDA-MB-231-bearing nude mice after various treatments for 21 d. Values represent means ± SD (n = 5).

**Figure S38.** Blood biochemistry indices (BUN, AST, ALT, and TP) of after various treatments for 21 d. Data are presented as the mean ± SD (n = 3).

References

[1] Y. Qin, X. Chen, Y. Gui, H. Wang, B. Z. Tang, D. Wang, *J. Am. Chem. Soc.* **2022**, *144*, 12825.
